# Essential Gene Pathways for Glioblastoma Stem Cells: Clinical Implications for Prevention of Tumor Recurrence

**DOI:** 10.3390/cancers3021975

**Published:** 2011-04-18

**Authors:** Kazunari Yamada, Jonathan Tso, Fei Ye, Jinny Choe, Yue Liu, Linda M. Liau, Cho-Lea Tso

**Affiliations:** 1 Department of Surgery/Surgical Oncology, David Geffen School of Medicine, University of California Los Angeles, 13-260 Factor building, 10833 Le Conte Avenue, Los Angeles, California 90095, USA; E-Mails: mdky@ucla.edu (K.Y.); crystaltofu@live.com (J.T.); feiy.los@ucla.edu (F.Y.); choe.jinny@gmail.com (J.C.); Yueliu@mednet.ucla.edu (Y.L.); ctso@mednet.ucla.edu (C.L.T.); 2 Department of Neurosurgery, David Geffen School of Medicine, University of California Los Angeles, Los Angeles, California, USA; E-Mail: lliau@mednet.ucla.edu (L.M.L.); 3 Jonsson Comprehensive Cancer Center, University of California Los Angeles, Los Angeles, California, USA

**Keywords:** glioblastoma, cancer stem cells, radial glial cells, neural crest cells, neural stem cells, radio-chemoresistance

## Abstract

Glioblastoma (World Health Organization/WHO grade IV) is the most common and most aggressive adult glial tumor. Patients with glioblastoma, despite being treated with gross total resection and post-operative radiation/chemotherapy, will almost always develop tumor recurrence. Glioblastoma stem cells (GSC), a minor subpopulation within the tumor mass, have been recently characterized as tumor-initiating cells and hypothesized to be responsible for post-treatment recurrence because of their enhanced radio-/chemo-resistant phenotype and ability to reconstitute tumors in mouse brains. Genome-wide expression profile analysis uncovered molecular properties of GSC distinct from their differentiated, proliferative progeny that comprise the majority of the tumor mass. In contrast to the hyperproliferative and hyperangiogenic phenotype of glioblastoma tumors, GSC possess neuroectodermal properties and express genes associated with neural stem cells, radial glial cells, and neural crest cells, as well as portray a migratory, quiescent, and undifferentiated phenotype. Thus, cell cycle-targeted radio-chemotherapy, which aims to kill fast-growing tumor cells, may not completely eliminate glioblastoma tumors. To prevent tumor recurrence, a strategy targeting essential gene pathways of GSC must be identified and incorporated into the standard treatment regimen. Identifying intrinsic and extrinsic cues by which GSC maintain stemness properties and sustain both tumorigenesis and anti-apoptotic features may provide new insights into potentially curative strategies for treating brain cancers.

## 1. Introduction

**Figure 1.**
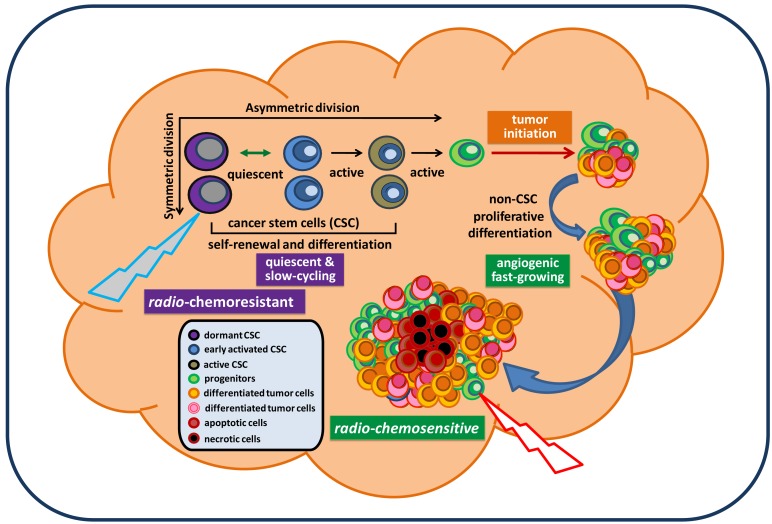
A theoretical model of a growing, treatment-resistant glioblastoma tumor. Glioblastoma stem cells (GSC) use both symmetric and asymmetric division to achieve sustained self-renewal and proliferative differentiation to initiate and maintain a tumor. GSC contain both quiescent and active cell types; the quiescent GSC are slow-cycling, poorly differentiated, radio-chemoresistant, and capable of unlimited self-renewal. The differentiated GSC progeny form a tumor containing a heterogeneous population in different states of differentiation, and are fast-growing, angiogenic, and radio-chemosensitive. GSC can escape from radio-chemotherapy and continually replenish tumor cells, leading to sustained tumorigenesis.

Cancer remains the third biggest killer worldwide. Although its survival rates are improving, it is still responsible for 13 percent of all deaths (according to the World Health Organization/WHO, 2010). To date, there is no approved effective treatment for relapsed advanced cancer. The mechanisms underlying persistent tumorigenesis and treatment resistance are still poorly understood, thereby making it difficult to develop an effective preclinical model for defining and predicting the efficacy of new treatments in patient trials. Among all human cancers, primary malignant brain tumors are one of the most challenging scientific and clinical problems in modern medicine. The tumor's location, its unique feature of high motility, and its protection by the blood brain barrier make certain therapies that are effective for some other cancers ineffective against brain tumors. In particular, glioblastoma remains virtually incurable despite extensive surgical excision and post-operative adjuvant radiotherapy and chemotherapy. The vast majority of glioblastoma patients will always develop tumor recurrence. The recent isolation and characterization of glioblastoma stem cells (GSC), a rare subpopulation within the tumor mass capable of unlimited self-renewal and sustenance of tumor growth, provides an excellent model to explain our inability to eradicate these tumors. Particularly, GSC isolated from recurrent glioblastoma tumors express molecular properties of a quiescent stem cell phenotype distinct from their proliferative progeny which make up the majority of tumor mass [1]. This suggests that they may evade standard cell cycle target-based therapy and continue seeding the new tumor, despite local treatment to the bulk tumor mass (Figure 1). Thus, identification of genes and pathways that confer the migratory ability, anti-apoptotic features, and tumorigenic capacity of GSC would be essential for a clear understanding of GSC and identifying potential targets in order to eradicate and prevent them from regenerating new tumors. This review summarizes signaling pathways that have been relatively well-studied in GSC and are essential for maintaining GSC stemness, tumorigenic potential, and anti-apoptotic features. Based on these reported signaling pathways relevant for maintaining GSC, we discuss and propose potential targeting strategies for future therapeutic developments in the treatment of brain cancer.

## 2. Molecular Pathways Associated with Tumorigenic Potential of GSC

**Figure 2.**
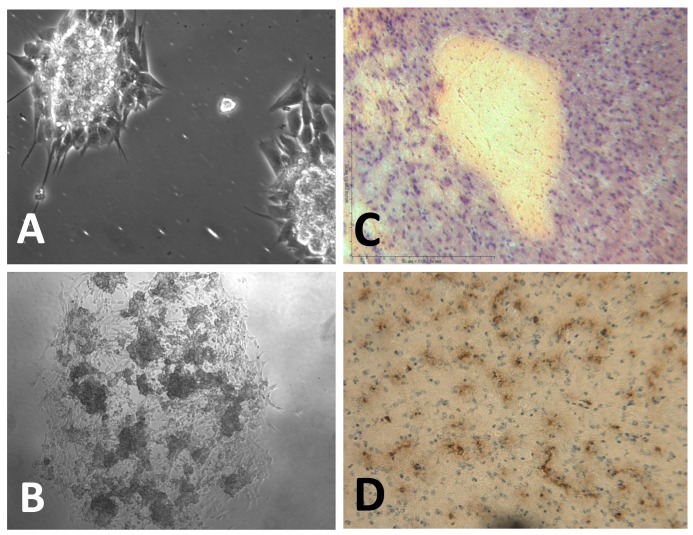
Glioblastoma stem cells (GSC) isolated from human tumor tissue are capable of forming tumor spheres in cultures and initiating a brain tumor in animal models. (**A**) GSC undergo continuous self-renewal and differentiation to populate tumor spheres in culture. (**B**) GSC are highly migratory cells, which can spontaneously migrate outward from primary tumor spheres, form the surrounding monolayer, and initiate secondary tumor spheres in culture. (**C**) Intracranial injection of GSC can lead to the development of infiltrating tumors *in vivo*. Hematoxylin and eosin (H-E) staining of tumors showed hypercellular zones surrounding necrotic foci, which form the histopathologic features of pseudopalisading necrosis-like glioblastoma as seen in human glioblastoma tumors. (**D**) Hypervascularity was evidenced by the strong positivity of CD31/PECAM-1 (platelet endothelial cell adhesion molecule-1), as determined by immuno histochemical staining.

Current experimental models for the study of GSC in the laboratory have been relatively standardized. At the functional level, they can persistently self-renew and differentiate, therefore sustaining tumorigenesis. In the laboratory, GSC are defined as patient tumor-derived glioblastoma cells expressing normal stem cell markers that are capable of clonal self-renewal and proliferative differentiation to populate tumor spheres in serum-free cultures supplemented with epidermal growth factor (EGF) and fibroblast growth factor (FGF) for indefinite passages (Figure 2A and 2B). Moreover, when these GSC are injected into animals, they are able to reconstitute glioblastoma tumors in mouse brains, which recapitulates the histopathological features of the patient tumor from which the GSC were derived (Figure 2C and 2D) [1-5]. By using these documented functions, which can be visualized, assayed, and quantitated, investigators have begun to explore more molecular signatures and gene pathways by which GSC are maintained and function. In particular, by use of loss-of-function phenotype studies, such as gene knockdown experiments, the role of a particular gene in maintaining GSC properties and function can be screened, determined and verified. Several surface molecules of GSC have been identified and used for GSC isolation and enrichment, including CD133/prominin [1,3], Musashi homolog 1 (MSI1) [6], and A2B5 [7,8]. Through studies in both *in vitro* and *in vivo* GSC functional models, several essential genes and signaling pathways for maintaining tumorigenic potential have been implicated.

### 2.1. Purified CD133+ GSC Derived from Treatment-Refractory Glioblastoma Tumor Express a Quiescent Phenotype

Although CD133/prominin is not an obligatory marker for GSC [6-9], CD133 was the first applied surface marker for the enrichment of GSC [1-5]. Indeed, multiple reports indicate that cancer stem cell expression may have prognostic value and that CD133 stem cell antigen expression affects clinical outcome in glioma patients [10-13]. In our recent study of GSC, we performed the first genome-wide expression profile analysis of purified, tumorigenic CD133+ GSC derived from treatment-refractory recurrent brain tumors [1]. We found that these tumorigenic CD133+ GSC possess characteristics of neuro ectoderm-like cells and express multiple markers for adult stem cells, including radial glial cells (RGC) (e.g., fatty acid binding protein 7, secreted protein acidic and rich in cysteine-like 1), neural stem cells (NSC) (e.g., SOX2, nestin), mesenchymal stem cells (MSC) (e.g., CD44, CD105), neural crest cells (NCC) (e.g., Distal-less homeo box 5/6, v-myc myelocytomatosis viral-related oncogene), and stem cells in the small intestine and colon (e.g., Leucine-rich repeat-containing G protein-coupled receptor 5). More importantly, we found that purified CD133+ GSC cells, not CD133+ glioblastoma spheres (containing mostly CD133- progeny), express a tumor-suppressor phenotype, which is characterized by the expression of a series of genes associated with a slow-growing, undifferentiated, polarized, migrating, anti-inflammatory, and anti-angiogenic phenotype [1]. These findings therefore imply that (i) CD133+ GSC cells may be clinically dormant/quiescent prior to undergoing proliferative cell division (PCD) to produce CD133- glioblastoma effector progeny, (ii) the dormant like phenotype may allow CD133+ GSC to escape from cell cycle-targeted radio-chemotherapy and regenerate new tumors, and (iii) genes guarding the pools and tumorigenic potential of GSC may not be in the subgroup of genes directly controlling cell proliferation, but in the subgroup regulating cellular quiescence, development, differentiation, and survival.

On the other hand, it was recently reported that the presence of proliferative CD133+/Ki67+ GSC positively correlated with disease progression and poor clinical outcome [14]. Meanwhile, the significance of CD133 as a GSC marker is being increasingly challenged. It appears that CD133 expression does not always mark GSC [4,6-9]. Moreover, the expression of CD133 on GSC seem to be subject to “microenvironments', depending on culture condition. Therefore, the GSC population is being considered a dynamic fraction of cells highly adaptable to microenvironmental changes. Exploring more definitive surface markers and signaling pathways specific to GSC in different stages (e.g., during tumor initiation, progression, and after treatment) would be essential for improving GSC isolation and better understanding GSC abilities, as well as developing new therapeutic targets. Thus, identifying and verifying intrinsic pathways and extrinsic cues by which GSC sustain self-renewal and anti-apoptotic features to support continuous tumor growth after standard treatments will facilitate the development of novel therapeutic strategies to diminish the recurrence rate of glioblastoma tumor.

### 2.2. Notch Signaling Pathway Maintains the Quiescent, Undifferentiating, and Tumorigenic Potential of GSC

It is plausible that cellular quiescence enables subsets of GSC to escape from cell cycle-based radio-chemotherapeutic treatment and re-enter the cell division cycle upon treatment removal, which leads to reinitiation of a new tumor. Notch signaling has been implicated in the maintenance of cellular quiescence in many adult stem cell pools by retaining self-renewal potential, inhibiting differentiation, and protecting them from exhaustion of their proliferative capacity [15-17]. Moreover, Notch signaling regulates NSC differentiation, and the induction of Notch signaling drives NSC into quiescence, whereas blocking Notch signaling reinitiates NSC division and neurogenesis [16,18]. The involvement of Notch signaling in cancer development was first indicated in T-cell acute lymphoblastic leukemias and lymphomas, which have activating mutations in the Notch 1 receptor, suggesting that Notch signaling may have a role in the maintenance of cancer stem cells [19]. Indeed, several Notch signaling pathway activation-associated genes, which may be linked to the regulation of reversibility of cellular quiescence, are determined to be overexpressed in purified CD133+ GSC [1] and have been described in NSC and brain tumor models [10,15-17]. These genes include inhibitor of differentiation 4 (ID4) [20], hairy and enhancer of split 1 (HES1) [15], hairy/enhancer-of-split related with YRPW motif 1 (HEY1) [21] and fatty acid binding protein 7 (FABP7) [22]. FABP7 is a direct target of Notch signaling in migrating RGC [23], which have been proposed to be a cellular origin of brain tumors [24], and increased expression of FABP7 was found to be associated with regions of glioblastoma tumor infiltration [25], suggesting that prolonged Notch activation in GSC may not only maintain the stemness of GSC, but also promote a migration and glial-fate specification [21,26,27].

The involvement of Notch signaling in the maintenance of the tumorigenic potential of GSC was indicated by the demonstration that treatment of glioblastoma sphere cultures with gamma-secretase inhibitors (GSIs) can deplete CD133+ GSC, downregulate putative GSC markers (CD133, nestin, BMI1, Olig2), and inhibit growth of tumor spheres and xenografts [23]. Investigators further concluded that Notch signaling blockade depletes tumorigenic GSC apparently through reduced cell proliferation and increased cell apoptosis associated with decreased levels of phosphorylated AKT and STAT3 [28]. Furthermore, a recent study showed a critical role for tumor endothelial cells in GSC maintenance, which is in part via Notch signaling and suggested that inhibition of Notch signaling in glioblastoma can target GSC via an endothelial cell intermediate. [29]. Thus, the targeted inactivation of Notch signaling could represent a novel, promising therapeutic strategy to cause GSC to dysfunction and become unable to regenerate a new tumor.

### 2.3. Hypoxia and Hypoxia-Inducible Factors Promote Self-Renewal and Survival of GSC and Regulate the Tumorigenic Capacity of GSC via Notch Signaling

It has been shown that hypoxia increases stem-like side population and CD133+ GSC [30]. GSC respond to hypoxia by activating hypoxia-inducible factor -1 alpha (HIF-1α) to enhance their self-renewal activity and anti-differentiated status [31]. Hypoxia requires Notch signaling for maintaining cells in an undifferentiated state, which occurs via activation of Notch-signaling target genes by recruiting HIF-1α to Notch-responsive promoters [32]. Likewise, HIF-2α and multiple HIF-regulated genes were reported to be preferentially expressed in GSC in comparison to non-stem tumor cells. The maintenance of GSC by a hypoxic microenvironment occurs partially via enhancing the activity of stem cell factors such as Oct4, c-Myc, and Nanog, thereby promoting and stabilizing the stem cell phenotype [33,34]. Functionally, loss of HIF-2α in GSC leads to a significant decrease in both GSC proliferation and self-renewal in cultures, and attenuation of tumorigenic capacity in animals [31]. Thus, HIFs might represent a promising target for eliminating GSC populations for a more effective treatment of glioblastoma.

### 2.4. Gli-Nanog Axis Promotes Stemness and Self-Renewal of CD133+ GSC and Glioblastoma Tumor Growth

It has been shown that Hedgehog (HH)-GLI signaling regulates the self-renewal and tumorigenicity of CD133+ GSC, and the blockade of HH by treatment with cyclopamine depletes stem-like cancer cells in glioblastoma [35]. Recently, pluripotency homeobox gene Nanog was characterized as a novel HH-GLI mediator essential for expanding CD133+GSC, maintaining a stemness phenotype, and promoting glioblastoma growth [36]. Nanog is regulated by HH-GLI signaling via binding of HH effectors, Gli1 and Gli2, to the Nanog promoter, thus activating Nanog expression [37]. Moreover, loss of p53, a tumor suppressor gene, promotes cell stemness and activates HH signaling, thereby contributing to Nanog upregulation. In contrast, p53 negatively regulates the activity and level of GLI1 and thus, downregulates Nanog expression [36-38]. The inhibitory loop between GLI1 and p53 is consistent with inversely reciprocal levels of GLI1 and p53, which have been shown in GSC culture [36]. Concomitantly, GLI1 upregulates Notch and downregulates BMP signaling, a pro-differentiative action on stem cells [38], implying a functional GLI1-NANOG-p53-Notch network in maintaining and regulating GSC function and fate.

### 2.5. Transforming Growth Factor Beta (TGFβ) Stimulates Self-Renewal, Inhibits Differentiation, and Promotes Tumorigenic Capacity of GSC via Activation of Leukemia Inhibitory Factor (LIF), Signal Transducers and Activators of Transcription 3 (STAT3), and Sry-Related HMG-box 2 (SOX2)

TGFβ is a pleiotropic cytokine and TGFβ/TGFβ receptor signaling by Smad proteins involves many cellular processes, including embryonal development, cell growth, differentiation, morphogenesis, wound healing, and immune regulation [39]. Alternatively, TGFβ signaling through Smad-independent pathways are known to activate Ras/extracellular signal-regulated kinase (ERK), TGFβ-activated kinase-1/p38 mitogen-activated protein kinase/c-Jun NH2-terminal kinase (TAK1/P38/JNK), phosphatidyl inositol 3-kinase(PI3K)/AKT, and STAT3 [40,41]. TGFβ signaling is known to promote tumor epithelial-mesenchymal transition (EMT), invasion, metastasis, and immune evasion, and the involvement of TGFβ-signal transduction in glioblastoma development from GSC has been suggested [42]. Indeed, a recent study indicated that TGFβ signaling promotes the self-renewal and tumorigenic capacity of GSC by induction of LIF through an activated Smad complex binding to the LIF promoter [43]. Moreover, treatment of GSC with recombinant LIF induced a rapid phosphorylation of STAT3, which is a downstream substrate of the LIF receptor complex. Thus, autocrine TGFβ signaling promotes GSC self-renewal through the activation of JAK-STAT pathway, and is mediated by the induction of LIF secretion [43]. Mice receiving GSC pretreated with a TGFβ receptor inhibitor and a JAK inhibitor exhibited a statistically significant increase in survival compared to that of the control group, indicating that inhibiting the TGFβ and JAK-STAT pathways decrease the self-renewal and tumorigenic potential of GSC [43].

STAT3 regulating the growth and self-renewal of GSC was further established by two studies, demonstrating that the direct inhibition of STAT3 signaling using a short hairpin RNA (shRNA)-mediated genetic knockdown of STAT3 or treatment with inhibitors of STAT3-DNA binding, leads to downregulation of stemness-associated genes, loss of capacity for tumor sphere formation, induction of cell apoptosis and differentiation, and a decrease in tumor-initiating capacity [44,45]. Moreover, since STAT3 signaling is a downstream effector of interleukin-6 (IL-6), blocking IL-6R alpha or IL-6 expression in GSCs by shRNAs suppresses tumor sphere formation capacity and increases the survival of mice bearing intracranial glioblastoma xenografts [46]. These data thus suggest that STAT3 signaling pathway may be a potential target for GSC-directed therapy of glioblastoma.

A recent study further indicated that TGFβ signaling maintains the tumorigenic capacity of GSC via induction of SOX2 expression, a stemness-associated gene, and such an induction was promoted by the expression of SOX4, which is a direct TGFβ target gene [47]. This study was further complemented by the demonstration of SOX2 silencing in GSC, leading to the loss of self-renewal capacity and tumorigenicity [48]. Moreover, induction of GSC differentiation by bone morphogenetic protein 4 led to the loss of tumorigenic capacity [49,50], indicating that maintaining the undifferentiated phenotype in GSC is one of the key criteria for retaining tumorigenic capacity.

### 2.6. Pro-survival AKT, MEK (Mitogen-Activated Protein Kinase/ERK Kinase), and ERK 1/2 Signaling from Epidermal Growth Factor Receptor (EGFR) Maintain Self-Renewal and Tumorigenic Capacity of GSC

It has been demonstrated that the EGFR signaling pathway is involved in the maintenance of GSC with enhanced malignant phenotypes [51]. The treatment of GSC with tyrosine kinase inhibitors of epidermal growth factor signaling suppresses self-renewal capacity and induces apoptosis of CD133+ GSC; the anti-proliferative effects of these drugs are likely mediated by the inhibition of phosphorylation of EGFR, AKT kinase, and ERK 1/2 [52,53]. A similar effect was observed by the inhibition of AKT activity in GSC using an AKT inhibitor, by which the survival of mice bearing human glioma xenografts was prolonged [54]. A recent study further showed that targeted inactivation of MEK/ERK signaling resulted in reduced sphere formation of GSC, accompanied by their differentiation into neuronal and glial lineages [55]. Moreover, combinational blockade of both MEK/ERK and PI3K/mTOR pathways suppressed self-renewal capacity and tumorigenic potential of GSC more effectively than blockade of either alone [56]. These results indicate that the maintenance of GSC requires EGF/EGFR signaling and its downstream effector activation, suggesting another molecular pathway target for an anti-GSC therapy in brain cancer.

### 2.7. c-Myc Is Required for Maintaining the Self-Renewal and Tumorigenic Potential of GSC

C-Myc is an oncogenic transcription factor that activates expression of a great number of genes through several mechanisms, including recruitment of histone acetylases and chromatin remodeling factors, as well as interaction with basal transcriptional factors [57]. C-Myc gene inactivation triggers telomere-independent senescence mediated by the cyclin-dependent kinase inhibitor p16INK4a, which is regulated by the polycomb group repressor Bmi-1, a direct transcriptional target of C-Myc [57]. C-Myc increases self-renewal in neural progenitor cells through Myc-interacting zinc-finger protein-1 (Miz-1) [58]. Likewise, c-Myc is highly expressed in GSC relative to non-stem glioma cells, and knockdown of c-Myc in GSC reduces cell proliferation, induces cell apoptosis, and causes loss of tumorigenic capacity [59]. Correspondingly, HIF-2α promotes GSC self-renewal and stemness properties by enhancing c-Myc [34], while inactivation of two tumor suppressor genes, PTEN and p53, leads to the increased expression of c-Myc and promotion of stemness, self-renewal and the tumorigenic capacity of GSC [60]. These data suggest that the c-Myc signaling pathway is required for maintenance of GSC, and c-Myc is an important target for the cooperative actions of p53 and PTEN in regulation of the tumorigenic potential of GSC.

### 2.8. Involvement of L1 Cell Adhesion Molecule (L1CAM), Olig2, BMI1, and Integrin α6 in the Maintenance of Growth, Survival, and Tumorigenic Capacity of GSC

L1CAM plays an important role in nervous system development, including neuronal migration and differentiation [61]. A recent study showed that L1CAM expression is preferentially higher in CD133+ GSC than in normal neural progenitors, and knockdown of L1CAM expression via shRNA interference inhibited the growth of CD133+ GSCs, disrupted sphere forming capacity, induced cell apoptosis, and suppressed tumor growth [62]. The induction of CD133+ GSC apoptosis by decreasing the expression of L1CAM is likely due to the decreased expression of the basic helix-loop-helix transcription factor Olig2 and the increased expression of the p21WAF1/CIP1 tumor suppressor [62]. It has been shown that an Olig2-regulated lineage-restricted pathway is critical for proliferation and maintenance of tumorigenic GSC through the suppression of p21WAF1/CIP1 [63].

BMI1 is an integral component of the polycomb repressive complex 1(PRC1), a complex required to maintain the transcriptionally repressive state of many genes by chromatin remodeling and histone modification. It has been shown that BMI1 is highly expressed in CD133+ GSC and stable BMI1 knockdown resulted in inhibition of self-renewal capacity, induction of both cell apoptosis and cell differentiation *in vitro*, and loss of tumorigenic capacity *in vivo* [64]. Likewise, disruption of EZH2, the main component of PRC2, strongly impairs GSC self-renewal *in vitro* and tumor-initiating capacity *in vivo* [65], suggesting that PcG proteins are required to sustain GSC self-renewal and maintain tumorigenic capacity.

Integrins are cell surface receptors that mediate developmental events by binding extracellular matrix ligands. Integrin α6 subunit is critical for the early development of the nervous system and has been shown to play a role in neural migration during olfactory development [66]. A recent study showed that GSC highly express integrin α6 and their interaction with laminin on endothelial cells directly regulates the tumorigenic capacity of GSC. Targeting integrin α6 in GSCs inhibits self-renewal, proliferation, and tumor formation capacity [67], suggesting that integrin α6 can be potentially used as a cellular target for targeting GSCs.

## 3. Molecular Pathways Linked to the Radio-Chemoresistant Phenotype of GSC

To date, temozolomide (TMZ; TEMODAR®) administered daily with radiation therapy (RT) for six weeks, followed by adjuvant TMZ for six months, has become the standard therapy for patients with newly diagnosed glioblastoma. In a large randomized phase III trial conducted in Europe and Canada, survival benefit was shown by adding TMZ to postoperative RT in the treatment of glioblastoma with five years of follow-up [68,69]. This study further showed that patients whose tumor had a methylated promoter for the gene encoding O^6^-methylguanine-DNA methyltransferase (MGMT) were more likely to benefit from the addition of TMZ [69,70]. Although the survival advantage of combined treatment lasts up to five years of follow-up, most patients successfully treated with combined therapy eventually had tumor recurrence and died [69]. An attempt to identify molecular profiles specific for treatment resistance to the concomitant radio-chemotherapy with TMZ in glioblastoma eluted a self-renewal signature, homeobox (HOX) genes, which include prominin-1 (CD133) [71]. Notably, tumors with the enhanced expression of HOX genes, high EGFR expression plus unmethylated MGMT were associated with short survival [72], suggesting the involvement of a tumor stem-cell phenotype in the escape of tumor cells from radio-chemotherapy.

Cellular quiescence is defined as a reversible growth/proliferation arrest, which is an essential property of many adult somatic stem cell populations and are usually regulated by tumor suppressor genes to maintain cell cycle arrest. Recent studies suggest that the quiescent stem cell nature adopted by cancer stem cells may explain the considerable resistance to chemotherapeutic agents [72-75]. Moreover, quiescent cells may show greater repair capacities than proliferative cells [74,76], suggesting that the nature of cellular quiescence in cancer stem cells may plays a key role in the acquired or constitutive resistance to radio-chemotherapy [77].

### 3.1. Activation of Checkpoint Proteins

Some studies have indicated that the presence of CD133+ cells correlates with glioblastoma malignancy and affects clinical outcome in glioma patients [10,78], suggesting that CD133+ GSC may play a major role in radio-chemoresistance and tumor aggressiveness. Indeed, it has been shown that CD133+ GSC can be enriched by radiation treatment in gliomas. CD133+ cells isolated from glioblastoma tumors preferentially activated the DNA damage checkpoint protein, Chk1 and Chk2 kinases [76], and repaired radiation-induced DNA damage more effectively than CD133- glioblastoma cells [76]. Moreover, the radioresistance of CD133+ GSC can be reversed by treatment with a specific inhibitor of the Chk1 and Chk2 checkpoint kinases, supporting the role of Chk1/2 kinases in radioresistance of GSC. Likewise, resistance of GSC to chemotherapeutic drugs has also been reported [71,79,80], suggesting that activation of the DNA damage checkpoint response or abnormalities of cell-death pathways may be the underlying mechanisms [81].

### 3.2. Evasion of Cell-Death Pathway

GSC exhibit enhanced chemoresistance to several chemotherapeutic agents [79,80,82]. It appears that the activity of the ATP-binding cassette transporter ABCG2 segregates a tumorigenic stem-like side population (SP) from non-stem-like cells [80], and TMZ treatment further increases this SP cells, and even more so when PTEN was deleted [80]. Moreover, MGMT expression is increased in SP cells, consistent with the resistance of SP cells to TMZ [80]. Likewise, several anti-apoptotic genes (e.g., BCL-2, BCL2L1a, and MCL1) were found to be at higher expression levels in TMZ resistant-GSC clones than those in differentiated cell lines [83]. In a separate study, a chemoresistant phenotype of CD133+ GSC was characterized by the enhanced expression of multidrug resistance 1 (MDR1) compared to CD133- non-stem cells [82]. Correspondingly, the radioresistance of GSC could be alleviated by treatment with an XIAP inhibitor [81], thus suggesting that the radio-chemoresistance of GSC may be linked to MGMT-mediated DNA repair and activation of both drug efflux transporters and anti-apoptotic factors.

### 3.3. Constitutively Active Notch and PI3K/Akt Signaling

Notch signaling is an essential pathway for maintaining stemness properties and tumorigenic potential of GSC [28]. Recent studies showed that blocking of Notch signaling by treatment with GSIs enhances the radiation-induced GSC death [84]. Moreover, the expression of the constitutively active intracellular domains of Notch1 or Notch2 in GSC attenuates the radiosensitizing effects of GSIs [84]. Notch signaling promotes radioresistance by upregulating PI3K/AKT pathway signaling and increasing the levels of a prosurvival Bcl-2 family member, myeloid cell leukemia-1 (MCL1). Importantly, the knockdown of Notch1 or Notch2 sensitizes GSC radiation therapy and impairs tumorigenic capacity [84], indicating a critical role of Notch/PI3K signaling in GSC radioresistance. Concordantly, addition of GSIs enhances TMZ treatment of human gliomas by inhibiting neurosphere repopulation and xenograft recurrence [85], pointing out the essential role of Notch pathway in chemoprotection of GSC.

### 3.4. BMI1-Mediated Recruitment of the DNA Damage Response Machinery

PcG protein complexes are mostly associated with heterochromatin, where they remodel chromatin such that epigenetic silencing of genes takes place [86]. BMI1 plays important roles in H2A ubiquitylation and Hox gene silencing, and is a potent negative regulator of the Ink4a/Arf locus, which encodes the cell cycle regulators and tumor suppressor p16Ink4a and p19Arf genes [87,88]. However, BMI1 is enriched in CD133+ GSC and required for maintaining GSC self-renewal in an Ink4a/Arf - independent manner [64]. BMI1 was enriched at the chromatin after irradiation and colocalized with ataxia-telangiectasia mutated (ATM) kinase and the histone gammaH2AX in glioblastoma cells, an important DNA double strand break (DSB) repair pathway [87]. Moreover, BMI1 preferentially copurified with non-homologous end joining (NHEJ) proteins in CD133+ GSC, suggesting that BMI1 confers radioresistance to GSC through the recruitment of DNA damage response machinery [88]. On the other hand, radiosensitive CD133+ GSC with a defective DNA damage response has been reported [89], and a separate study also did not find different DNA repair mechanisms in stem and non-stem cells [90].

### 3.5. Insulin-Like Growth Factor Binding Protein 2 (IGFBP2)-Mediated Activation of AKT Signaling

IGFBP2 is known to be overexpressed in a majority of glioblastoma tumors, and its expression is inversely correlated to glioblastoma patient survival [91,92]. It has been reported that IGFBP2 enhances invasion by upregulating invasion-enhancing proteins such as matrix metalloproteinase-2 and CD24 [93,94]. Recent studies indicated that IGFBP2 is overexpressed in GSC [1,95] and autocrine IGFBP2 is required for self-renewal and expansion of GSC [95].The knockdown of IGFBP2 expression depleted the expression of stemness-associated genes and reduced AKT activation, while treatment with an IGFBP2 neutralizing antibody sensitized GSC to irradiation and multiple antineoplastic agents [95]. Furthermore, recombinant IGFPB2 substantiates AKT signaling-mediated GSC viability that could be blocked by treatment with PI3K/Akt inhibitors. These data thus suggest that IGFBP2 mediates a protective effect against DNA-damage agents, thus contributing to GSC chemoresistance.

## 4. Models for Targeting the Mechanisms of Radio-Chemoresistance within Glioblastoma Stem Cell Pathways

**Figure 3.**
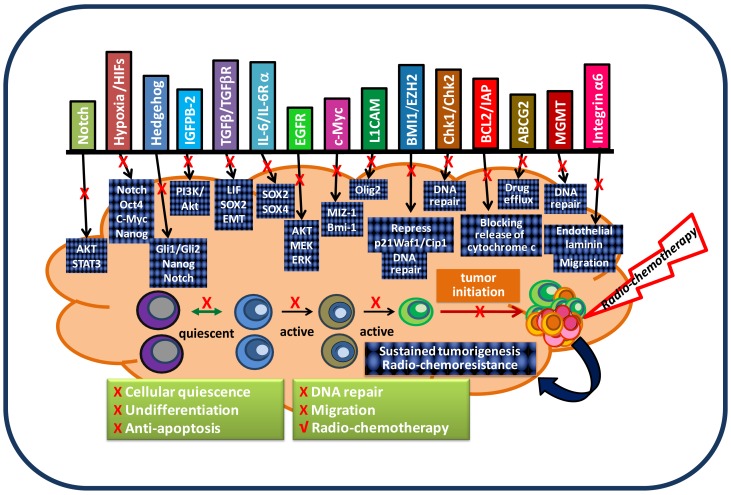
A model of glioblastoma stem cell (GSC)-targeted therapy. GSC have the ability to self-renew as well as give rise to new tumors. In order to prevent post-treatment tumor recurrence, a treatment targeting essential gene pathways for GSC must be incorporated into current therapeutic modalities. Several gene pathways have been determined to be required for maintaining tumorigenic capacity and a radio-chemoresistant phenotype of GSC. These signaling pathways work collaboratively and cooperatively to generate a quiescent, undifferentiated, and anti-apoptotic phenotype as well as to constitutively activate the DNA damage checkpoint response in GSC. Importantly, GSC exhibit a chemoresistant phenotype via constant activation of the DNA damage checkpoint response and AKT survival pathway, as well as expression of high levels of both anti-apoptotic proteins and drug efflux transporters. In order to eradicate a tumor, a therapeutic strategy that disrupts GSC signaling pathways must be developed to be fully integrated into radio-chemotherapy in order to target both GSC and non-GSC populations.

Because the cancer stem cell (CSC) hypothesis, models, and molecular pathways are not yet fully established, unknown molecular targets and essential pathways for maintaining tumorigenic capacity and radio-chemoresistance will continue to be discovered. These accumulated preclinical data will certainly facilitate the development of new concepts in tumor biology and the design of potentially more effective treatment protocols for preventing radio-chemoresistant CSC-mediated tumor recurrence. In the mean time, it is important to note that normal CD133+ neural stem/progenitor cells are also recruited by recurrent tumors and their relative percentage favorably affects the survival of patients [13]. Hence, there is the possibility that targeting new pathways may also eliminate normal neural stem/progenitor cells, given their dependence on the same signaling pathways as cancer stem cells. Exploring differences between normal and tumor stem cells may reveal novel molecular targets for a safe therapy for brain cancer. Based on the molecular pathways discussed in this paper, a therapeutic model for targeting both fast-growing, non-stem-like tumor cells and slow-cycling, chemoresistant GSC is proposed and displayed in Figure 3.

## 5. Conclusions

Laboratory evidence indicates that glioblastoma tumors contain a small population of neural stem-like cells capable of clonal self-renewal to form tumor spheres in cultures and initiate/repopulate a tumor in animal models. GSC express unique cellular, molecular, and functional properties distinct from their proliferative differentiating progeny. GSC utilize multiple stem cell signaling pathways to achieve a radio-chemoresistant phenotype that sustains tumorigenesis. Radio-chemoresistance may be accomplished via collaboration of: (i) constitutive activation of the DNA damage checkpoint response and PI3K-Akt signaling pathway, (ii) high expression of both anti-apoptotic proteins and drug efflux transporters, and (iii) evasion of both differentiation and irreversible cell cycle arrest. Moreover, it is clear that as intrinsic properties of GSC continue to be better characterized, identifying the extrinsic cues from their niche that effect GSC is also crucial as they may provide vital signaling to modulate GSC physiology and pathology [96,97]. The cure for cancer requires eliminating both GSC and non-GSC populations. Thus, in order to develop a more potent and effective brain cancer therapy, it is important to design preclinical studies and clinical trials which evaluate the synergistic benefits of incorporating GSC-targeted therapies into conventional cancer treatments.
